# *Polygonum capitatum*, the Hmong Medicinal Flora: A Comprehensive Review of Its Phytochemical, Pharmacological and Pharmacokinetic Characteristics

**DOI:** 10.3390/molecules27196407

**Published:** 2022-09-28

**Authors:** Yan Lin, Lei He, Xing-Jun Chen, Xu Zhang, Xue-Long Yan, Bo Tu, Zhu Zeng, Ming-Hui He

**Affiliations:** 1 Key Laboratory of Infectious Immune and Antibody Engineering of Guizhou Province, Engineering Research Center of Cellular Immunotherapy of Guizhou Province, Guizhou Medical University, Guiyang 550025, China; 2 School of Pharmacy, Guizhou Medical University, University Town, Guian New District, Guizhou 550025, China; 3 State Key Laboratory of Functions and Applications of Medicinal Plants, Guizhou Medical University, Guiyang 550031, China

**Keywords:** *Polygonum capitatum*, flavonoids, pharmacological activities, chemical analysis

## Abstract

*Polygonum capitatum*, known as “Tou Hua Liao” (Chinese name), is a crucial source of Hmong medicinal plants that has benefited human health for a long time. This folk-medicinal plant is widely distributed in the south-west of China for the treatment of various urologic disorders including urinary tract infections, pyelonephritis, and urinary calculus. The purpose of this paper was to provide a systematic and comprehensive overview of the traditional usages, botany, phytochemistry, pharmacology, pharmacokinetics and clinical applications of this flora. Up until the end of 2022, at least 91 compounds had been reported from *P. capitatum*, mainly covering the classes of flavonoids, lignanoids, phenols and other components. The compounds and extracts isolated from *P. capitatum* exhibit a wide range of pharmacological activities, such as anti-inflammatory, antioxidant, antimicrobial, anticancer, analgesic, hypothermic, diuretic and other pharmacological effects. Qualitative and quantitative chemical analyses were also covered. Furthermore, the possible development trends and perspectives for future research on this medicinal plant were also discussed.

## 1. Introduction

*Polygonum capitatum* is a well-known and large-scale Miao medicinal plant with a long history of economic and medicinal value. Amongst Chinese people, especially in Guizhou province, *P. capitatum* is commonly named TouHuaLiao [1]. It is often named *Gastrochilus panduratum* RIDL., *Kaempferia pandurata* Roxb., *Curcuma rotunda* L., and *Boesenbergia rotunda* Linn [2]. At present, *P. capitatum* shows a variety of pharmacological activities, including anti-inflammatory, antioxidant, antimicrobial, anticancer, analgesic, hypothermic, diuretic and other pharmacological effects [2,3,4,5,6,7]. These pharmacological activities are attributed to the chemical constituents and extracts of *P. capitatum*. So far, more than 90 compounds have been isolated from *P. capitatum*. Phenolic acids and flavonoids are believed to be responsible for the bioactivities of *P. capitatum*.

With recently increasing levels of research into *P. capitatum*, it is especially valuable to review its current status in order to provide reference for a deep exploration of its ethnic medicinal potential. This review summarized progress in the chemical study of *P. capitatum*, mainly covering the classes of flavonoids, lignanoids, phenols, and other components. Moreover, we systematically organized the development of the medicinal flora into traditional usages, botany, and pharmacology. Qualitative and quantitative chemical analyses were also covered. Furthermore, a number of *P. capitatum*-based drugs (Relinqing Granule and Milin Capsule) have been approved by the State Food and Drug Administration. The other *P. capitatum*-based drugs, Relinqing^®^ Granule and Milin^®^ Capsule, have also been approved by the China Food and Drug Administration [3]. In addition, research directions for the future and prospects of *P. capitatum* were also discussed in this article.

### 1.1. The Traditional and Ethnical Uses of P. Capitatum in China

*P. capitatum* first appeared in the “*Guangxi Traditional Chinese Medicine Annals*”, as a method to dispel wind, disperse blood stasis and relieve pain [8]. Contemporary among the works of “*Guangxi Chinese Herbal Medicine*”, it possessed the effects of detoxification and inflammation, and was chiefly used for the treatment of dysentery, skin ulcers, and unspecified poisonous swelling [9]. Furthermore, it was recorded in the masterworks of “*Yunnan Chinese Herbal medicine*”; it had the pharmacological effect of clear heat diuresis drenching [10]. The literature was consistent with the present *P. capitatum*. In the 2010 edition of the Chinese Pharmacopoeia (Appendix), the name of the medicinal material was *Polygonum capitatum* Buch. -Ham. ex D. Don was given as the name of the herb [11]. The medicinal parts were collected as whole dry herbs or aerial parts. *P. capitatum* exhibits heat- and damp-clearing functions as a medicinal diuretic [1]. Moreover, in folk medicine it was frequently decocted with water, the therapeutic effect was more obvious, but it was inconvenient to take [1]. In recent years, various pharmaceutical factories in Guizhou province have made full use of and developed *P. capitatum* resources, together with their diversified preparation products that have appeared successively. Among them, Relinqing Granule is the most representative prescription, and has the functions of heat-clearing and detoxifying, diuresis and dredging stranguria. They are used for hot stranguria caused by damp-heat in the lower energizer [12].

### 1.2. Botanical Description

*P. capitatum,* is derived from the dried herbs of Polygonaceae species (Polygonaceae family), and is a perennial herb, 10–15 cm tall, with stolons, rooting at its nodes, annual branches ascending upward, and a red surface. Leaves are alternately oval-, base-, and wedge-shaped, sometimes with “V”-shaped markings on the leaf surface, inflorescent, terminal, perianth reddish, five-parted, and flowering from June to October each year. It is worth noting that the stem of the transverse section is composed of one to two rows of epidermis cells. The outermost part has glandular hairs where the cortex is narrow and composed of many rows of tightly arranged parenchyma cells that contain more clusters of calcium oxalate crystals. In addition, the leaf of *P. capitatum* is a heterofacial leaf. That is to say, the upper epidermis of the leaf of the main vein protrudes slightly upward, semicircularly, and the parenchyma cells of the protruding part are small and dense with glandular hairs often growing in the lower epidermis. Furthermore, the vascular bundles in the middle of the main veins are arranged in a ring; they are externally tough vascular bundles. Parenchyma cells are round, scattered calcium oxalate clusters. The palisade tissue is generally composed of two to three rows of long oval cells, which are closely arranged with scattered clusters of calcium oxalate crystals [13,14].

### 1.3. Geographic Distribution

*P. capitatum* is generally distributed in the southwest of China, mainly in Jiangxi, Guizhou, Hunan, Hubei, Sichuan, Guangxi, Yunnan, and Tibet provinces. It is also found in other Asian countries, including India, Nepal, Bhutan, Myanmar and Vietnam. The plants of *P. capitatum* are fond of growing in cool and moist places, they are suitable for growing in the sandy loam of sunny valleys with good air permeability and mild acidic soil without water shortages [13,14]. 

## 2. Phytochemistry

To date, there are a total of 91 compounds (**1**–**91**) with the phytochemical composition of *P. capitatum*. They can be classified into four classes: 30 flavonoids, 10 lignanoids, 25 phenols, and 26 other compounds. Each phytochemical is numbered (**1**–**91**) and their names, formulas, molecular weights, and the parts of plant used in these compounds, are cited in the SI (Supportting Information) (Table S1).

### 2.1. Flavonoids

**Figure 1:**
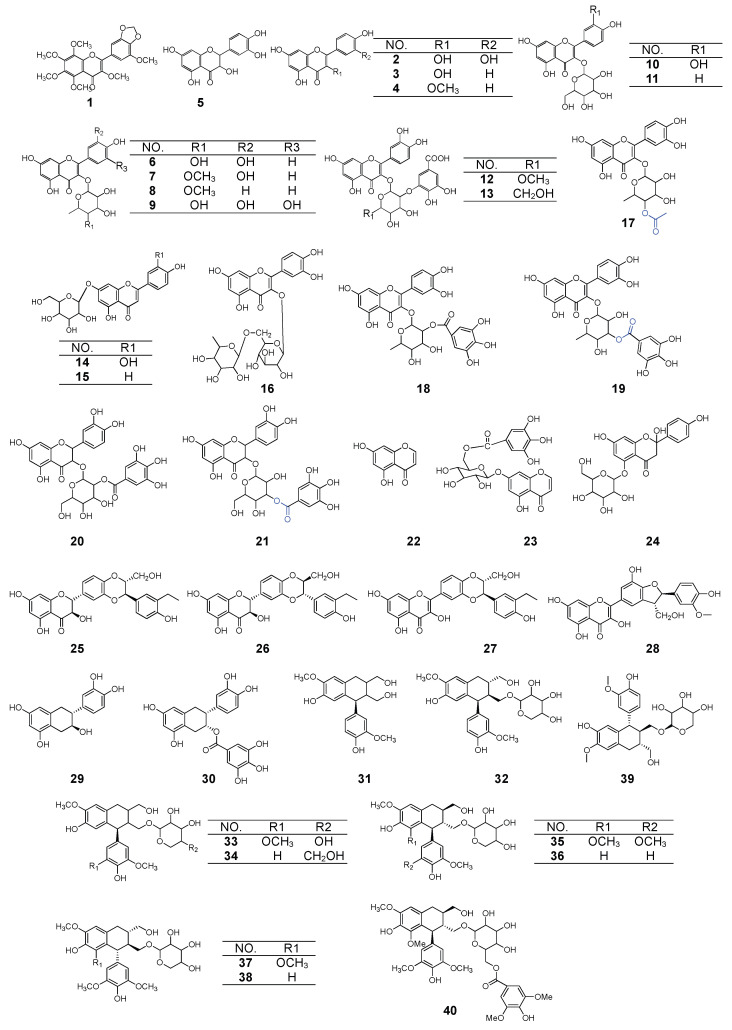
The chemical structure of compounds from *P. capitatum*.

Flavonoids are large secondary metabolites found in *P. capitatum*. More than 30 flavonoid compounds from *P. capitatum* have been isolated and their structures confirmed. The main flavonoids are flavanones, flavones, flavonol glycosides, and dihydroflavone alcohol glycosides. However, some of these flavonoids also exist in other plants. Most of them show a unique structure with an acylated monosacchride residue integrated in their main skeleton. Thirty flavonoids have been separated from *P. capitatum* and their chemical structures are displayed in Figure 1. In 2001, 3’,4’-methylenedioxy-3,5,6,7,8,5’-hexamethylflavone (**1**) [5] was first isolated from *P. capitatum*, which is an unusual flavone of *P. capitatum* [11]. After that, quercetin (**2**) [15], kaempferol (**3**) [6], kaempferol-3-methyl ether (**4**) [16], and taxifolin (**5**) [16] were separated and purified from *P. capitatum*. Furthermore, the flavonoids and their glycosides are a widespread occurrence in *P. capitatum*. Among them, glycosylation at C-3 of the nucleus has been found to be the most commonly present, and rhamnose, glucose, arabinose, and rhamnosyl-rhamnose are the most common sugars found as glycones of this flavonol glycoside, including quercitrin (**6**) [16], quertin-3-O-(4’’-methoxy)-α-L-rhamnopyranosyl (**7**) [16], kaempferol-3-O-α-L-rhamnopyranoside (**8**) [15], myricetrin (**9**) [17], hirsutrin/quercetin-3-O-β-D-glucopyranoside (**10**) [15], kaempferol-3-O-β-D-glucopyranoside (**11**) [15], 2’’-O-galloyl quercitrin (**12**) [15], 2’’-O-galloyl hirsutrin (**13**) [15], luteoloside/luteolin-7-O-glucoside/cymaroside (**14**) [7], daidzin (**15**) [18], rutin (**16**) [17], quercetin-3-O-(4’’-O-acetyl)-α-L-rhamnoside (**17**) [17], quercetin-3-O-α-L- rhamnoside-2’’-gallte (**6**) [17], quercetin-3-O-α-L-rhamnoside-3’’-gallate (**19**) [19], quercetin-3-O-(2’’-O-rhamnoside)-β-D-glucopyranoside (**20**) [19], querctin-3-O-(3’’-O-rhamnoside)-β-D-glucopyranoside (**21**) [19], 2,7,4’-trihydroxyflavanone-5-O-β- D-glucopyranoside (**24**) [20], and epicatechin-3-O-gallate (**30**) [19]. Most of these were separated from *P. capitatum* for the first time. In particular, some new styles of flavonol glycoside (**12**–**13**, **18**–**21**) [15,17,19], combined with a substituent of the gallic acid group, were first isolated from it, which may play an important role in their pharmacoactivity. Meanwhile, a new chromone glycoside (7-O-(6-galloyl)-β-D-glucopyranosyl-5-hydroxychromone (**23**) [21] and one known chromone (5,7-dihydroxychromone (**22**) [22] were isolated. Moreover, the isolation of four flavonoid lignans of silymarin, for which the structure was the condensation of flavanonol and phenyl propanoid derivatives, including silybin (**25**) [16], isosilybin (**26**) [16], 2,3-dehydrosilybin (**27**) [16] and 2,3-dehydrosilychristin (**28**) [16] from *P. capitatum,* were new styles of lignans. In addition, a common flavanone catechin (**29**) [16] was found from this plant.

### 2.2. Lignanoids

Ten lignanoids were isolated and identified from *P. capitatum*. The structures of these compounds are shown in Figure 1. Moreover, it belongs to isolariciresinol (**31**) [16], (+)−isolariciresinol-3a-O-β-dxylopyranoside (**32**) [16], (+)-5’-Methoxyisolariciresinol-9-O-β-D-Xylopyranoside (**33**) [16], (+)−isolariciresinol-3a-O-β-D-glucopyranoside (**34**) [16], nudiposide (+) lyoniresinol 3α-O-β-D-xylopyranoside (**35**) [16], isolariciresinol-2a-O-β-D-xylopyranoside (**36**) [17], lyoniside/(-) lyoniresinol-3α-O-β-D-xylopyranoside (**37**) [23], 5’-methoxyisolariciresinol-2a-O-β-D-xylopyranoside (**38**) [24], schizandriside (**39**) [24], and lyoniresinol-2a-O-[6-O-(4-hydroxy-3,5-dimethoxy)-benzoyl]-β-D-glucopyranoside (**40**) [24]. For them, nudiposide and (+) lyoniresinol 3α-O-β-D-xylopyranoside, lyoniside and (-) lyoniresinol 3α-O-β-D-xylopyranoside, are the same compound, respectively. Furthermore, (+)−isolariciresinol-3a-O-β-dxylopyranoside/isolariciresinol-2a-O-β-D-xylopyranoside and nudiposide/lyoniside are two pairs of absolute configuration, and were isolated and identified from the herbs of *P. capitatum*.

### 2.3. Phenolics

**Figure 2:**
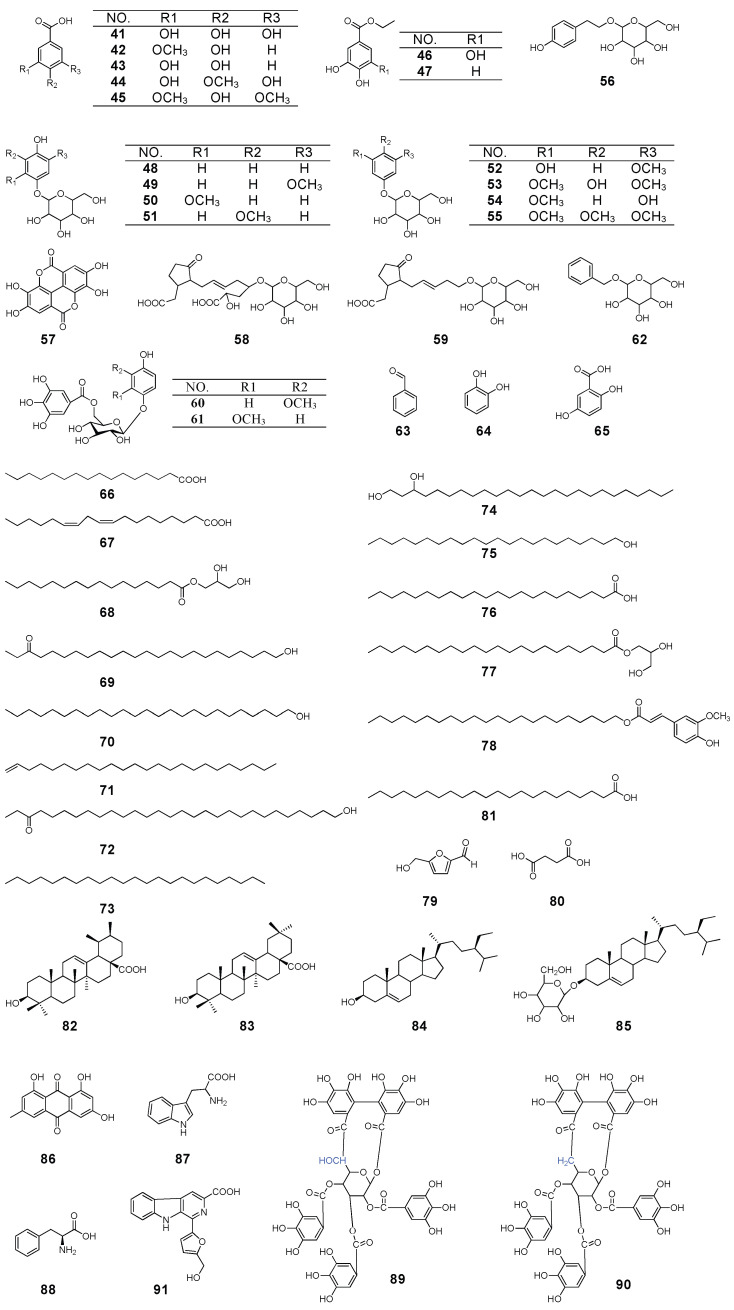
The chemical structure of compounds from *P. capitatum* (continued).

According to the literature, phenolic compounds are the secondary abundant constituents in *P. capitatum.* So far, a total of 25 phenolic compounds (**41**–**65**) have been separated from this plant (Figure 2, Table S1). Among them, gallic acid (**41**) [25], vanillic acid (**42**) [24] and protocatechuic acid (**43**) [24] are the major ones and have been confirmed to possess various pharmacological activities. Moreover, 2-methoxyl-1,4-benzenediol-1-O-β-D-glucopyranoside/2-methoxy-4-hydroxyphenol-1-O-β-D-glucopyranoside/isotachioside and 1,3-dimethoxyl-2,5-benzenediol-5-O-β-D-glucopyranoside/3,5-dimethoxy-4-hydro-xyphenol-1-O-β-D-glucopyranoside, were the same compounds, respectively. It should be noted that, phenolic glycosides (**48**–**56**, **58**–**62**) [19,22,24] were reported for the first time from *P. capitatum* and the family Polygonaceae.

### 2.4. Other Compounds

Other compounds have also been isolated and identified from *P. capitatum*, and their structures are shown in Figure 2. Sixteen organic acids, alcohols, esters and aldehydes, including palmitic acid (**66**) [26], linoleic acid (**67**) [26], hexadecanoic acid-2,3-dihydroxypropyl ester (**68**) [26], 24-hydroxy-24-alkane-3 (**69**) [24], pentacosanol (**70**) [26], 28 alkyl-1,27-diene (**71**) [26], 29-hydroxy-29-alkanone-3 (**72**) [26], tricosane (**73**) [24], behenic acid (**74**) [26], tricosanol (**75**) [26], lignoceric acid (**76**) [26], docanoic acid -2,3-dihydroxypropyl ester (**77**) [26], docosyl ferulate (**78**) [26],5-hydroxymethylfurfural (**79**) [27], succinic acid/butanedioic acid (**80**) [26] and tetracosane-1,3-diol (**81**) [26] were identified from the petroleum ether extracts of *P. capitatum*. Furthermore, four terpenoids have been isolated from this ethnic medicine, including ursolic acid (**82**) [16], oleanolic acid (**83**) [16], β-sitosterol (**84**) [28] and β-daucosterol (**85**) [28]. Only one anthraquinone component, emodin (**86**) [16], has been separated from it. Of note, 1,5,7-trihydroxy-3-methylanthraquinone (Yu was isolated from *P. capitatum* in 2008) and emodin were found to be the same component. Two amino acids have been identified from the *n*-butanol fraction of the ethanol extract of *P. capitatum*, including L-tryptophan (**87**) [20] and L-phenylalanine (**88**) [22]. Quite recently, two ellagitannins, davidiin (**89**) [29] and FR429 (**90**) [30], were discovered from it. In addition, one alkaloid, flazine (**91**) [28], was also identified in *P. capitatum*.

## 3. Biological Activities and Medicinal Potential

As a folk medicine, the whole of the *P. capitatum* plant has been used to treat urinary tract infections, dysentery, eczema, urolithiasis and pyelonephritis by the Hmong residents from China. It has long been conceived that gallic acid is the only composition underwriting the pharmacological effects of *P. capitatum*. However, the anti-inflammatory effect of *P. capitatum* extract has been ascribed to gallic acid-free fractions abounding in flavonoids. Thus, the phenolics and flavonoids are both considered as crucial bioactive constituents of *P. capitatum*.

**Figure 3:**
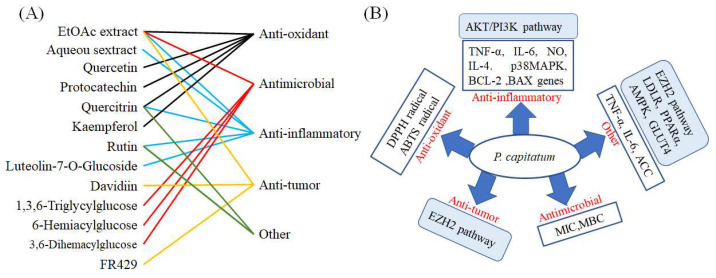
The bioactivities (**A**) and proposed mechanisms (**B**) of *P. capitatum* compounds.

Plenty of investigations have been reported on the pharmacological activities of *P. capitatum* extracts and its major compounds. In the past two decades, pharmacological studies on *P. capitatum* have indicated diverse biological activities, including anti-inflammatory, antioxidant, anti-hepatocellular carcinoma, antibacterial, antitumor, analgesic, hypothermic, and diuretic activity. This research is summarized here with special focus on flavonoids and phenolic acids with medicinal potential (Figure 3, Table 1).

### 3.1. Anti-Inflammatory Activities

The pharmacological effects of *P. capitatum* on anti-inflammatory activity have been fully summarized. The aqueous and ethanol extract of *P. capitatum* exhibits anti-inflammatory effects by inhibiting the levels of inflammatory cytokines NO and TNF-α in RAW 264.7 macrophages [31]. The largest study was reported by Liao, the total flavonoid fractions were tested on Kunming mice (18–22 g), administrated orally through gavage in a single dose of 0.6 g/kg, 0.3 g/kg, and 0.15 g/kg per day for seven consecutive days. The results showed significant anti-inflammatory activity with inhibition rates of 86.15 % at 0.6 g/kg [32]. Furthermore, treatment with flavonoid-rich extract of *P. capitatum* (the major constituents were luteolin-7-O-glucoside, rutin, and quercitrin) at 90 and 180 mg/kg body weight in rats for 6 weeks remarkably decreased serum TNF-α, and interleukin-6 (IL-6) levels, which mechanism implied that total flavonoids suppressed the development of atherosclerosis, possibly by inhibiting inflammatory response [7]. Later, the anti-inflammation effects of total flavonoids of both wild and cultivated *P. capitatum* were also observed in mouse abdominal cavity capillary permeability, the xylene-induced ear swelling model and carrageenan-induced mouse pedal swelling test, and the results showed an inhibitory effect in the same dose [33]. To screen effective anti-inflammatory extracts from *P. capitatum*, they reported that the aqueous extract and the protein-free water extract of *P. capitatum* could significantly inhibit the release of NO, TNF-α and IL-6 in LPS-induced RAW264.7 cells. In particular, the protein-free water extract of *P. capitatum* had the best effect on NO, TNF-α and IL-6 inhibition and was the main effective anti-inflammatory ingredient [34]. Recently, quercetin, one flavonoid, was isolated from *P. capitatum*, and regulated the balance of gastric cell proliferation and apoptosis to protect against gastritis. Its mechanism was that quercetin protects against gastric inflammation and apoptosis associated with *Helicobacter pylori* infection by affecting the levels of p38MAPK, BCL-2 and BAX genes [35]. At the same time, flavonoid glycosides of *P. capitatum* protect against inflammation associated with *Helicobacter pylori* infection, and the results suggested that flavonoid glycoside has repairing functions for gastric injuries [36].

In addition, the *P. capitatum* extract powder (1.58 g/kg body weight, DW) in CMC-Na solution, was orally administered for SD rats once daily for 14 consecutive days. The results proved *P. capitatum* could inhibit the activation of the AKT/PI3K pathway by upregulating PTEN expression; thus, gastric mucosal inflammation induced by H. pylori can be improved [37]. *P. capitatum* has a significant therapeutic effect on allergic contact dermatitis, which may be related to suppression of levels of IL-4 and TNF-α [38]. In particular, Relinqing granules (14.4, 7.2 g/kg DW) promisingly inhibited dimethylbenzene-induced auricle tumefaction of mice. Relinqing granules (3.6, 7.2 g/kg DW) significantly inhibited granuloma with cotton ball in rats. Relinqing granules (7.2 g/kg DW) significantly decreased the number of white blood cells in rat urine with chronic urinary tract infections, and improved kidney function and pathological changes [39].

The search for a better model system to explore the effective constituents and the mechanism of action of anti-inflammatory *P. capitatum* was studied through the method of network pharmacology. The results showed a total of 6 active compounds, and 41 potential targets and 76 signal pathways were screened and obtained [40].

### 3.2. Anti-Oxidant Activities

The anti-oxidant activities of *P. capitatum* and its flavonoids have been studied extensively using different anti-oxidant models. These models were induced 2-20-azinobis-3-ethylben-zthia zoline-6-sulphonate (ABTS), 1,1-diphenyl-2-picrylhydraz-yl (DPPH), hydrogen peroxide (H_2_O_2_). The proposed situation/mechanisms are summarized in Table 1.

*P. capitatum* extract has demonstrated obvious anti-oxidant activity in vitro. Experimental studies have shown that an 80% methanol extract of leaves and stems from *P. capitatum* demonstrate strong antioxidant activities against ABTS^+^/OH^−^ (23.08%) and Fe^2+^ chelating capacity activities (17.3% EDTA/g DW) [41]. Some flavonoids isolated from *P. capitatum*, quercitrin, protocatechuic acid, quercetin and kaempferol possessed strong scavenging free radical capacity against H_2_O_2_, with an IC_50_ of 0.044 μM, 0.276 μM, 0.098 μM and 0.029 μM, respectively [42]. For in vitro experiments, the ethanol extract revealed stronger anti-oxidant activities than the aqueous extracts of *P. capitatum*; its IC_50_ values were 1.71 mg/mL and 0.15 mg/mL, respectively [43]. The same result was shown in another study; the methanol extract of *P. capitatum* showed higher scavenging activity against DPPH radical and ABTS radical Particularly, the methanol extract exhibited more significant antioxidant activity than that of positive drug BHT [44]. In addition, the EtOAc extract of *P. capitatum* exhibited remarkable scavenging activity against DPPH radical and ABTS radical. The results further elucidate that EtOAc extract could be used as an important part of antioxidant substances, and that polyphends were the major active ingredients of antioxidant activity for *P. capitatum* [58]. However, it is of great importance to note that only a small part of the research conducted into anti-oxidant activity has employed in vitro based methods and that further in vivo verifications should be encouraged.

### 3.3. Antimicrobial Activities

**Figure 4:**
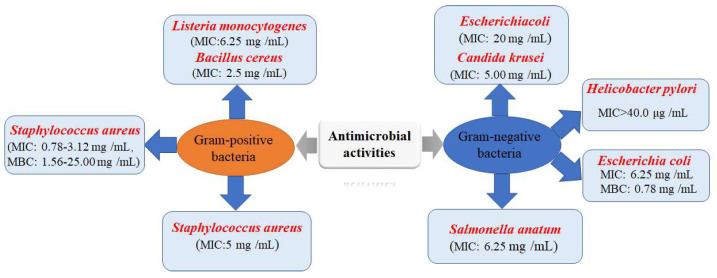
MICs and MBCs (mg/mL) of the extracts of *P. capitatum* against bacterial strains.

Plenty of investigations have been reported on the antimicrobial activities of *P. capitatum* extracts, and the major compounds *P. capitatum* possesses and their promising antibacterial activities (Figure 4). Previous studies have reported that crude extracts of *P. capitatum* significantly inhibit the growth of the bacteria *Listeria monocytogenes* and *Salmonella anatum*, at the minimum inhibitory concentration (MIC) of 6.25 mg/mL [41]. Liu et al. reported that the 60% ethanol extract (250 μg/disc) displayed a better antibacterial activity against the multidrug-resistant *Staphylococcus aureus* [46]. Moreover, in another study, plant extracts and fractions of *P. capitatum* demonstrated antimicrobial properties against bacterial strains, and through the determination of the MIC and the minimum bactericidal concentration (MBC), the results showed that the crude extracts or fractions FV (flavonoid-enriched fraction) and TN (tannin-enriched fraction) have antibacterial and bactericidal properties [32]. Additionally, in an in vitro antibacterial test, 40 μg/mL or higher concentrations of extracts (flavonoid glycosides) of *P. capitatum* inhibited the growth of *H. pylori*; the resistance of MIC was regarded as >40.0 μg/mL, while the resistance of MIC of amoxicillin was regarded as >1.0 μg/mL [36].

Simultaneously, *P. capitatum* inhibits *H. pylori* growth via interfering with and inhibiting the expression of *Helicobacter pylori* protein [47]. Moreover, four effective parts of the alcohol extract of *P. capitatum* were found to have outstanding potential antimicrobial activities; the main antibacterial components could be 6-galacyl glucose, 3, 6-digalacyl glucose, 1, 3, 6-trigalacyl glucose and Davidiin [48]. Moreover, the different polar of seven fractions in the 70% ethanol extract of *P. capitatum* had high antibacterial activity against *EScherichia coli*, the MIC was 0.20 mg/mL, and the MBC was 0.78 mg/mL [49]. These findings show that antimicrobial activity is an essential property of *P. capitatum* and that this flora should be a fundamental source of preservatives for the pharmaceutical industry.

### 3.4. Anti-Tumor Activities

Some pharmacological studies have shown that different extract and compound prescriptions derived from *P. capitatum* have significant antineoplastic effects against diseases. In 2013, Wang et al. showed that emodin at doses of 10–120 mΜ could effectively inhibit production with a dose-dependent manner of HCC cell lines. The possible mechanism of action inhibited the expression of the proteasome-dependence of EZH2 [29]. It was also found that intraperitoneal administration (single dose of 10 mg/kg/day, sp) significantly inhibits tumor progression in hepatoma xenograft mice [30]. It is well known that davidiin displays extensive antitumor activity. Davidiin, a natural product isolated from *P. capitatum*, has an antitumor mechanism of changing the metabolism of sphingolipids. When HepG2 cells were treated with 50 μM davidian for 72 h, the levels of several types of sphingolipids significantly changed, including Cer, LacCer and So; they decreased markedly to 26.2%, 27.8% and 19.7%, respectively [50].

### 3.5. Other Biological Activities

Apart from anti-inflammatory, anti-oxidant, antimicrobial and anti-tumor activities, *P. capitatum* has a remarkable effect on anti-atherosclerosis, a hypoglycemic effect, and defervescence and analgesic action. Wang et al. reported that luteolin-7-O-glucoside, rutin and quercitrin total flavonoids, separated from *P. capitatum*, exerted an anti-atherosclerosis effect in hyperlipidemia rats through regulating blood lipid metabolism, and modulating a proinflammatory profile [7]. At the same time, the lignans (isoidulinol, 5’-methoxy-isolaridosin-9-O-β-D-xylopyranoside) isolated from *P. capitatum* showed significant hypoglycemic activity against type two diabetes [51]. Later, it was reported that aqueous extract of *P. capitatum* at a dose of 450 mg/kg DW significantly reduced the body temperature of rabbits with a fever induced by an intravenous injection of typhoid fever and *Paratyphoid bacillus* [52]. Furthermore, the alcohol and water extracts of *P. capitatum* exhibited a prominent analgesic effect on the writhing response induced by acetic acid in mice [33,53]. In addition, *P. capitatum* extracts (5 g/kg, 10 g/kg, 20 g/kg DW, 4 weeks) demonstrated a hypoglycemic effect. This mechanism may be related to the expression of AMPK and GLUT4 genes up-regulated in the liver to further promote the uptake of glucose by the liver tissue [54].

## 4. Quality Control

LC/MS or HPLC are currently the most powerful techniques for global chemical analysis of TCM. They have been extensively used for the analysis of chemical constituents of *P. capitatum*. The previous literature has reported flavonoids and phenolic acids were considered to be the vital active constituents of *P. capitatum*. In the 2003 edition of the “Quality standards of Chinese medicinal materials and ethnic medicinal materials in Guizhou Province”, only gallic acid (the content > 0.05%) was included as a standard for the evaluation of *P. capitatum* quality [45]. Zhang et al. reported an HPLC method to analyze the herbs of *P. capitatum*; the average content of gallic acid was 0.2% [59]. Over the past few years, the use of reversed-phase HPLC has been developed for the analysis of flavonoids; quercitrin, derived from *P. capitatum*, was linear and ranged from 0.082–0.408 μg [60]. In 2010, a scientist established a simple HPLC method for the characterization of quercetin from three parts (flower, stem and leaf) of *P. capitatum*. The results showed that the quercetins ranged from 0.25% to 0.62%, and the highest content of quercetin was found in leaves [61]. Recently, the Beijing Institute of Materia Medica, Chinese Academy of Medical Sciences, has completed the quality standard of *P. capitatum*. The content of gallic acid and quercetin should not be less than 0.015 g/100 g DW and 0.1 g/100 g DW, respectively [62].

## 5. Pharmacokinetic and Metabolite Analysis

**Figure 5:**
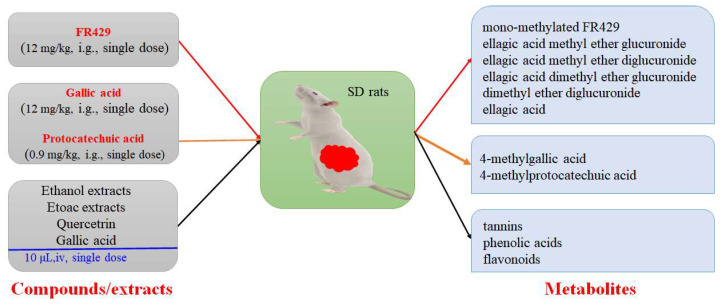
The pharmacokinetic and metabolite analysis of compounds from *P. capitatum*.

A comparative pharmacokinetic study of crude herb from *P. capitatum* was carried out. Several research groups have studied the metabolism of gallic acid (GA) and protocatechuic acid (PCA) in the aqueous extract of *P. capitatum*. Administration of aqueous extract of *P. capitatum* was at oral doses of 60 mg/kg (equivalent to 12 mg/kg DW of GA and 0.9 mg/kg DW of PCA) to rats; after 1 h, the concentration of GA and PCA in kidney tissue, respectively, reached 1218.62 ng/g and 43.98 ng/g, indicating that extensive metabolism of GA and PCA occurred after ingestion [3]. He et al. studied the material metabolism of the bioactive extracts of *P. capitatum*. The results showed that the metabolic pathways of intestinal flora in *P. capitatum* were hydrolysis, reduction and oxidation [55]. After that, the metabolic characteristics of FR429 were evaluated in male Wistar rats (260–280 g), a total of eight metabolites were detected from bile and urine. It was deduced that the main metabolic pathway of FR429 in rats was methylation and subsequent glucuronidation [56]. Recently, the extract of *P. capitatum* 700 mg/kg DW (equivalent to gallic acid 21.35 mg/kg DW, quercetin 2.17 mg/kg DW, quercetin content of 0.392 mg/kg DW, respectively,) was orally administered to rats. As a result, gallic acid and quercitrin were detected in plasma, but quercetin was not detected [2]. Similarly, ultra-high performance liquid chromatography-tandem mass spectrometry (UPLC-MS/MS) was used to determine the plasma levels of *P. capitatum* extracts. Compared with the normal group, the absorption of GA, PCA and quercetin (QR) in pyelonephritis rats was increased, and excretion was decreased [57] (Figure 5).

## 6. Conclusions

*P. capitatum* is a traditional medicinal plant of the Miao people and has been used to treat a variety of urological disorders in China over a long history, such as dysentery, pyelonephritis, cystitis, urolithiasis, pelvic inflammation and rheumatic pain. In this work, we reviewed the available information concerning the traditional uses, phytochemistry, pharmacology and quality control of *P. capitatum*. In total, 91 compounds from *P. capitatum* were isolated, including 30 flavonoids, 10 lignanoids, 25 phenols, and 26 other constituents. Furthermore, *P. capitatum* has clear pharmacological properties such as antibacterial, anti-inflammatory, antioxidant, antitumor, antipyretic and analgesic effects, and has potential hypoglycemic development prospects. These research results could provide a referential merit for the processing, quality control and clinical medication guidance of *P. capitatum*. In addition, some drugs have been derived from *P. capitatum* and are presently used in clinic such as Relinqing granule and Milins capsules, but the development of its related medical products is still very limited. However, it is also necessary to further study the drug-forming properties and pharmacokinetics of the active constituents of *P. capitatum*, as well as to establish quality control standards for different areas of *P. capitatum*, to investigate their safety evaluation, adverse reactions and toxicity, and to carry out research at the cellular and molecular levels. We hope that this review highlights the important value of *P. capitatum* and promotes its all-round development.

## Figures and Tables

**Table 1:** The bioactivities of extracts and components from *P. capitatum* (PC).

Pharmacological Activity	Extract/Compound	Model	Test Living System	Routes of Administration/Dose	Measured Parameters and Results	Ref.
Anti-inflammatory	aqueous and eth- anol extract of PC	In vitro	RAW 264.7 cells	1.6–250 mg/mL	NO, TNF-α (*p* < 0.05)	[31]
	total flavonoids of PC	In vivo	KM mice	i.g/0.6, 0.3,0.15g/kg/once daily/7 days	ear edema RLI: 86.15%	[32]
	total flavonoid of PC	In vivo	SD rats	i.g/90, 180 mg/kg/two daily/42 days	IL-6, TNF-α	[7]
	total flavonoids of PC	In vivo	KM mice	i.g/30, 60 g/kg/once daily/5 days	ear edema RLI: 39.26%	[33]
	total flavonoids of PC	In vivo	KM mice	i.g/30, 60 g/kg/once daily/5 days	TAMA (*p* < 0.05)	[34]
	total flavonoids of PC	In vivo	KM mice	i.g/30, 60 g/kg/once daily/5 days	granulmat (*p* < 0.05)	[34]
	aqueous extract of PC	In vivo and vitro	RAW 264.7 cells, SD rats	i.g/6 g/kg/two daily/4 days	NO (*p* < 0.01)	[35]
	protein-free aque- ous extract of PC	In vivo and vitro	RAW 264.7 cells, SD rats	i.g/6 g/kg/two daily/4 days	TNF-α, IL6 (*p* < 0.05, *p* < 0.01)	[35]
	quercetin	In vitro	GES-1 cells	8, 16, 32, 64, 128, 256, 512, 1024 µg/mL	p38MAPK, BCL-2 and BAX (*p* < 0.05)	[36]
	flavonoid glycosides	In vivo	C57BL/6 mice	i.p/32,64,128 µg/three daily/9 days	IL-4, IFN-γ, gastrin (*p* < 0.05)	[37]
	PC extract powder	In vivo	mSD rats	i.g/1.58 g/kg/once daily/14 days	AKT, PTEN, PI3K (*p* < 0.05)	[37]
	PC extract powder	In vivo	Balb/c mice	i.g/2.5, 5, 10%/once daily/7 days	IL-4, TNFα (*p* < 0.05)	[38]
	Relinqing Granule	In vivo	KM mice	i.g/7.2,14.4 g/kg/once daily/7 days	ear edema RLI: 59.28%	[39]
	Relinqing Granule	In vivo	SD rats	i.g/3.6, 7.2 g/kg/once daily/7 days	WBC count (*p* < 0.05)	[40]
	Relinqing Granule	In vivo	SD rats	i.g/7.2 g/kg/once daily/28days	granulmat (*p* < 0.01)	[41]
antioxidant						
	the 80% methanol extract of PC	In vitro	ABTS^·+^/OH^−^	8 mg/mL	(Radical scavenging activities: 23.08%)	[42]
	flavonoids and phenolic acids in PC	In vitro	O_2_^-^, OH, H_2_O_2_	395 g	O_2_^−^, OH, H_2_O_2_ (IC_50_: 1.149, 0.098,4.766 μg/mL)	[43]
	the 95% ethanol extract of PC	In vitro	OH, DPPH·	15 mg/mL	OH, DPPH· IC_50_:1.71 mg/mL	[44]
	PC extracts	In vitro	DPPH, ABTS, FRAP	3.5 mL/2.85 mL/3.8 mL	DPPH, ABTS (IC_50_: 2.98, 2.54 μg/mL)	[45]
	the extract of polyphenols from PC	In vitro	DPPH, OH^-^, ABTS	0.08 mg/mL	DPPH, OH^-^, ABTS	[41]
antibacterial						
	the 80% methanol extract of PC	In vitro	Listeria monocytogenes and Salmonella anatum	MIC 6.25 mg/mL		[46]
	the 60% ethanol extract of PC	In vitro	MIC, MBC	250 μg/disc		[32]
	the water and 70% aqueous ethanol of PC	In vitro	MIC, MBC	0.6 g/kg		[36]
	*P. capitatum* extracts	In vitro	MIC	40 μg/mL		[47]
	*P. capitatum* aqueous solution	In vitro	MIC	4 mg/mL		[48]
	the 35% methanol extract of PC	In vitro	Inhibition zone diameter	10 μL		[49]
Antitumor						
	davidiin	In vivo	HCC cells/male athymic nude mice	i.g/10 mg/kg/21 days	metablites (methylation and sulfate metabolites) cell viability (IC_50_: 60.9 μM)	[29]
	FR429	In vitro	Protein concentratio- ns	3 mg/mL	cell viability (IC_50_: 59.0 μM)	[30]
	davidiin	In vitro	HepG2 cells/SPL	5 mg/mL		[50]
Other biological activities						
	the whole plants of PC	In vivo	SD rats	i.g/45,90, 180 mg/kg/once daily/42 days	PPARα, LDLR mRNA (*p* < 0.01)	[7]
	dried whole grass of PC	In vitro	α-amylase	500 μL	α-amylase RLI: 146.1%	[51]
	aqueous extract of PC	In vivo	rabbit	0.45 g/kg, 0.01 g/kg/once daily/7 days	Temperature (*p* < 0.01, *p* < 0.001)	[52]
	PC extract powder	In vivo	KM mice	0. 2 mL/10 g/once daily/5 days	CP (*p* < 0.05)	[33]
	the whole plants of PC	In vivo	KM mice	20 g/kg, 20 mL/kg,0.2 mL	UV (*p* < 0.05)	[53]
	aqueous and eth- anol extract of PC	In vivo	male db/db mice	i.g/5, 10, 20 g/kg/once daily/42 days	OGTT, SOD, IL-6, INS (*p* < 0.05)	[54]
Metabolite analysis						
	gallic acid and protocatechuic acid	In vivo	SD rats	12 mg/kg,0.9 mg/kg/60 min	metabolites (4-OMeGA, 4-OMePCA)	[3]
	The ethanol and ethyl acetate extracts of PC	In vivo	SD rats	i.g/2, 20, 37.5, 37.5, 50 mL	Metabolites (22 metabolites)	[55]
	FR429	In vivo	Male SD rats	12 mg/kg,10 mg/mL/once daily/42 days	Metabolites (8 metabolites)	[56]
	gallic acid, quercitrin and quercetin	In vivo	Male SD rats	60 mg/kg	extraction rate (94.3–98.8%, 88.9–98.8%,95.7–98.5%)	[2]
	gallic acid, protoca -techuic acid and quercitrin	In vivo	Female SD rats	10 g/kg/once daily/3 days	extraction rate (87.18%)	[57]

## Supplementary Materials

The following supporting information can be downloaded at: https://www.mdpi.com/article/10.3390/molecules27196407/s1, Table S1: The compounds isolated from *P. capitatum*.
