# Supplementary material for: Polygonum capitatum, the Hmong Medicinal Flora: A Comprehensive Review of Its Phytochemical, Pharmacological and Pharmacokinetic Characteristics
Source: Molecules. 2022 Sep 28;27(19):6407. doi: 10.3390/molecules27196407 (PMC9571880; doi:10.3390/molecules27196407)
Supplement: Supplementary file 1 [file molecules-27-06407-s001.zip › molecules-1904366-supplementary.pdf]

Polygonum capitatum, the hmong medicinal flora: A comprehensive review of its phytochemical, pharmacological and pharmacokinetic characteristics

Yan Lin[1,2,3, Δ], Lei He[1,2,3, Δ], Xing-Jun Chen [1,2,3], Xu Zhang[1,2,3], Xue-Long Yan[1,2,3], Yi-Tong Shen[1,2,3], Zhu Zeng[1,2,3*], Bo Tu[1,2,3*] and Ming-Hui He[1,2,3*]

[1] Key Laboratory of Infectious Immune and Antibody Engineering of Guizhou Province, Engineering Research Center of Cellular Immunotherapy of Guizhou Province, Guizhou Medical University, Guiyang 550025, China;
[2] School of Pharmacy, Guizhou Medical University, University Town, Guian New District, Guizhou, 550025, China;
[3] State Key Laboratory of Functions and Applications of Medicinal Plants, Guizhou Medical University, Guiyang, China.
* Correspondence: authors：heminghui@gmc.edu.cn (M. H. He); tb3318@gmc.edu.cn (B.T.); zengzhu@gmc.edu.cn (Z Z.); Tel./fax: +86 0851-88416149
[Δ] The first two authors contributed equally to this work.

Table S1 The compounds isolated from P. capitatum.

No.	Structure type and chemical name	Molecular formula	Isolation Part	References
**Flavonoids**				
**1**	3',4'-methylenedioxy- 3,5,6,7,8,5'-hexamethylflavone	$C_{22}H_{22}O_{10}$	w	[16]
**2**	quercetin	$C_{15}H_{10}O_7$	w	[17]
**3**	kaempferol	$C_{15}H_{10}O_6$	w	[18]
**4**	quercetin 3-methyl ether	$C_{16}H_{12}O_7$	w	[19]
**5**	taxifolin	$C_{15}H_{12}O_7$	w	[19]
**6**	quercitrin	$C_{21}H_{20}O_{11}$	w	[19]
**7**	quercetin-3-O-(4''-methoxy)-α-L-rhamnopyranosyl	$C_{22}H_{22}O_{11}$	w	[19]
**8**	kaempferol-3-O-α-L-rhamnopyranoside	$C_{22}H_{22}O_{10}$	w	[17]
**9**	myricetrin	$C_{21}H_{20}O_{12}$	w	[20]
**10**	hirsutrin/quercetin-3-O-β-D-glucopyranoside	$C_{21}H_{20}O_{12}$	w	[17]
**11**	kaempferol-3-O-β-D-glucopyranoside	$C_{21}H_{20}O_{11}$	w	[17]
**12**	2''-O-galloyl quercitrin	$C_{28}H_{24}O_{15}$	w	[17]

**13**	2''-O-galloyl hirsutrin	$C_{28}H_{24}O_{16}$	w	[17]
**14**	luteoloside/luteolin-7-O-glucoside/cynaroside	$C_{21}H_{20}O_{11}$	w	[21]
**15**	daidzin	$C_{21}H_{20}O_{9}$	w	[22]
**16**	rutin	$C_{27}H_{30}O_{16}$	w	[20]
**17**	quercetin-3-*O*-(4''-*O*-acetyl)-*α*-L-rhamnoside	$C_{23}H_{22}O_{12}$	w	[20]
**18**	quercetin-3-O-*α*-L-rhamnoside-2''-gallate	$C_{28}H_{24}O_{15}$	w	[20]
**19**	quercetin-3-O-*α*-L-rhamnoside-3''-gallate	$C_{28}H_{24}O_{15}$	w	[23]
**20**	quercetin-3-O-(2''-O-rhamnoside)-β-D-glucopyranoside	$C_{28}H_{26}O_{16}$	w	[23]
**21**	quercetin-3-O-(3''-O-rhamnoside)-β-D-glucopyranoside	$C_{28}H_{26}O_{16}$	w	[23]
**22**	5,7-dihydroxychromone	$C_{9}H_{6}O_{4}$	w	[26]
**23**	7-O-(6′-galloyl)-β-D-glucopyranosyl-5-hydroxychromone	$C_{22}H_{20}O_{13}$	w	[25]
**24**	2,7,4' -trihydroxyflavanone-5-O-β-D-glucopyranoside	$C_{21}H_{22}O_{11}$	w	[24]
**25**	silybin	$C_{26}H_{24}O_{9}$	w	[19]
**26**	isosilybin	$C_{26}H_{24}O_{9}$	w	[19]
**27**	2,3-dehydrosilybin	$C_{26}H_{22}O_{9}$	w	[19]
**28**	2,3-dehydrosilychristin	$C_{25}H_{20}O_{10}$	w	[19]
**29**	catechin	$C_{16}H_{16}O_{5}$	w	[19]
**30**	epicatechin-3-O-gallate	$C_{23}H_{20}O_{9}$	w	[19]
**Lignanoids**				
**31**	isolariciresinol	$C_{20}H_{24}O_{6}$	w	[19]
**32**	(+)-isolariciresinol-3a-O-β-dxylopyranoside	$C_{25}H_{32}O_{10}$	w	[19]
**33**	(+)-5' -Methoxyisolariciresinol-9-O-β-D-xylopyranoside	$C_{26}H_{34}O_{11}$	w	[19]
**34**	(+)–isolariciresinol-3a-O-β-D-glucopyranoside	$C_{26}H_{34}O_{10}$	w	[19]
**35**	Nudiposide/(+)lyoniresinol 3*α*-O-β-D-xylopyranoside	$C_{27}H_{36}O_{12}$	w	[19]
**36**	isolariciresinol-2a-O-β-D-xylopyranoside	$C_{25}H_{32}O_{10}$	w	[20]
**37**	lyoniside/(-)lyoniresinol3*α*-O-β-D-xylopyranoside	$C_{37}H_{46}O_{17}$	w	[27]
**38**	5'-methoxyisolariciresinol-2a-O-β-D-xylopyranoside	$C_{25}H_{32}O_{10}$	w	[28]
**39**	schizandriside	$C_{27}H_{36}O_{12}$	w	[28]
**40**	lyoniresinol-2a-*O*-[6-*O*-(4-hydroxy-3,5-dimethoxy)-benzoyl]-*β*-*D*-glucopyranoside	$C_{26}H_{34}O_{11}$	w	[28]
**Phenolics**				

**41**	gallic acid	$C_7H_6O_5$	w	[29]
**42**	vanillic acid	$C_8H_8O_4$	w	[28]
**43**	protocatechuic acid	$C_7H_6O_4$	w	[28]
**44**	3,5-dihydroxy-4-methoxybenzoic acid	$C_8H_8O_5$	w	[24]
**45**	4-hydroxy-3,5-dimethoxybenzoic acid / syringate	$C_9H_{10}O_5$	w	[24]
**46**	ethyl gallate	$C_9H_{10}O_5$	w	[19]
**47**	ethylprotocatechuate	$C_9H_{10}O_4$	w	[19]
**48**	arbutin	$C_{12}H_{16}O_7$	w	[19]
**49**	2-methoxyl-1,4-benzenediol-4-O-β-D-glucopyranoside	$C_{13}H_{18}O_8$	w	[19]
**50**	2-methoxy-4-hydroxyphenol-1-O-β-D-glucopyranoside /isotachioside/2-methoxyl-1,4-benzenediol-1-O-β-D-glucopyranoside	$C_{13}H_{18}O_8$	w	[24]
**51**	3-methoxy-4-hydroxyphenol-1-*O*-β-D-glucopyranoside /tachioside	$C_{13}H_{18}O_8$	w	[24]
**52**	5-methoxyl-1,3-benzenediol-1-O-β-D-glucopyranoside	$C_{13}H_{18}O_8$	w	[24]
**53**	3,5-dimethoxy-4-hydroxyphenol-1-O-β-D-glucopyranoside/1,3-dimethoxyl-2,5-benzenediol-5-O-β-D-glucopyranoside	$C_{14}H_{20}O_9$	w	[19]
**54**	3-methoxy-5-hydroxyphenol-1-O-β-D-glucopyranoside /picraquassioside D	$C_{13}H_{18}O_8$	w	[19]
**55**	3,4,5-trimethoxyphenol--1-O-β-D-glucopyranoside	$C_{15}H_{22}O_9$	w	[19]
**56**	salidroside	$C_{14}H_{20}O_7$	w	[19]
**57**	ellagic acid	$C_{14}H_6O_8$	w	[22]
**58**	β -D-glucopyranosyl-12-hydroxy-12-(2'' -hydroxy-2'' -carboxyethyl) jasmonic acid	$C_{21}H_{32}O_{12}$	w	[24]
**59**	β -D-glucopyranosyl-12-hydroxyjasmonic acid	$C_{18}H_{28}O_9$	w	[24]
**60**	3-methoxy-4-hydroxyphenol-1-O-β-D-(6' -*O*-galloyl) glucopyranoside	$C_{20}H_{22}O_{12}$	w	[30]
**61**	2-methoxy-4-hydroxyphenol-1-O-β-D-(6'-*O*-galloyl) glucopyranoside	$C_{20}H_{22}O_{12}$	w	[24]
**62**	benzyl-*O*-β-D-glucopyranoside	$C_{13}H_{18}O_6$	w	[24]
**63**	benzaldehyde	$C_7H_6O_1$	w	[26]
**64**	catechol	$C_6H_6O_2$	w	[20]
**65**	2,5-dihydroxybenzoic acid	$C_7H_6O_4$	w	[26]

**Others**				
**66**	palmitic acid	$C_{16}H_{32}O_2$	w	[31]
**67**	linoleic acid	$C_{18}H_{32}O_2$	w	[31]
**68**	hexadecanoic acid-2,3-dihydroxypropyl ester	$C_{19}H_{38}O_4$	w	[31]
**69**	24-hydroxy-24- alkane-3	$C_{24}H_{48}O_2$	w	[28]
**70**	Pentacosanol	$C_{25}H_{52}O$	w	[31]
**71**	28 alkyl -1,27- diene	$C_{28}H_{54}$	w	[31]
**72**	29-hydroxy-29-alkanone -3	$C_{29}H_{58}O_2$	w	[31]
**73**	tricosane	$C_{23}H_{48}$	w	[28]
**74**	behenic acid	$C_{22}H_{44}O_2$	w	[31]
**75**	tricosanol	$C_{22}H_{46}O$	w	[31]
**76**	lignoceric acid	$C_{22}H_{44}O_2$	w	[31]
**77**	docanoic acid -2,3- dihydroxypropyl ester	$C_{25}H_{50}O_4$	w	[31]
**78**	docosyl ferulate	$C_{32}H_{54}O_4$	w	[31]
**79**	5-hydroxymethylfurfural	$C_6H_6O_3$	w	[32]
**80**	succinic acid/butanedioic acid	$C_4H_6O_4$	w	[32]
**81**	tetracosane-1,3-diol	$C_{25}H_{52}O_2$	w	[31]
**82**	ursolic acid	$C_{30}H_{48}O_3$	w	[19]
**83**	oleanolic acid	$C_{30}H_{48}O_3$	w	[19]
**84**	β-sitosterol	$C_{29}H_{50}O_1$	w	[33]
**85**	β-daucosterol	$C_{35}H_{60}O_6$	w	[33]
**86**	emodin/1,5,7-trihydroxy- 3-methylanthraquinone	$C_{15}H_{10}O_5$	w	[19]
**87**	L-tryptophan	$C_{11}H_{12}N_2O_2$	w	[24]
**88**	L-Phenylalanine	$C_4H_4N_2O_2$	w	[30]
**89**	davidiin	$C_{41}H_{30}O_{26}$	w	[34]
**90**	FR429	$C_{41}H_{30}O_{25}$	w	[35]
**91**	flazine	$C_{17}H_{12}N_2O_4$	w	[33]

Note.w: whole grass
